# Supplementary material for: Targeted NanoBiT Screening Identifies a Novel Interaction Between SNAPIN and Influenza A Virus M1 Protein
Source: Biology (Basel). 2025 Dec 11;14(12):1770. doi: 10.3390/biology14121770 (PMC12730228; doi:10.3390/biology14121770)
Supplement: Supplementary file 1 [file biology-14-01770-s001.zip › Supplementary Figure S1.pdf]

Supplementary Figure S1

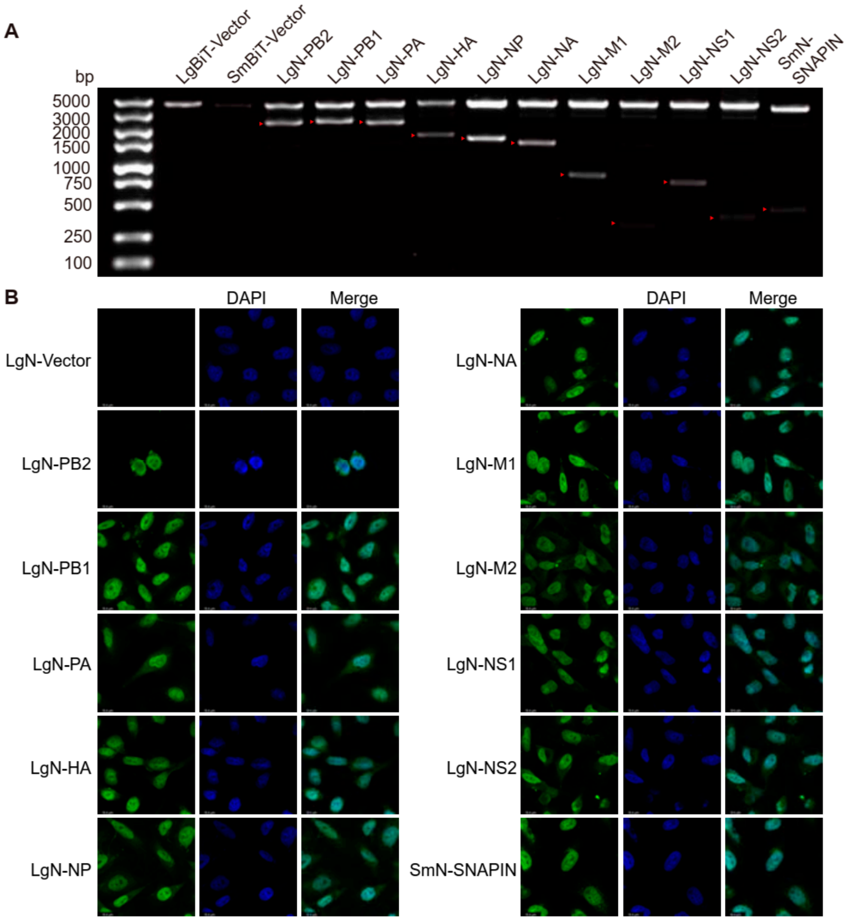

Supplementary Figure S1. Validation of plasmid integrity and expression efficiency. Double-restriction digestion of plasmids (A). LgBiT-Vector, SmBiT-Vector, LgN-PB2, LgN-PA, LgN-NA, LgN-M1, LgN-M2, LgN-NS1, LgN-NS2, and SmN-SNAPIN were digested using EcoRI and XbaI; LgN-PB1 and LgN-HA were digested using XhoI and XbaI; LgN-NP was digested using EcoRI and NheI. From lanes 3 to 12, the red triangles mark the insert fragments generated by double digestion, which match the expected sizes for PB2 (2280 bp), PB1 (2274 bp), PA (2151 bp), HA (1698 bp), NP (1497 bp), NA (1362 bp), M1 (759 bp), M2 (294 bp), NS1 (693 bp), NS2 (366 bp), and SNAPIN (411 bp). Confocal microscopy (B). A549 cells were transfected with the indicated plasmids. At 26 hpt, cells were fixed with 4% paraformaldehyde in PBS for 15 min and permeabilized with 0.5% Triton X-100 in PBS for 30 min. After blocking with 5% BSA in PBS for 1 h, cells were incubated with anti-LgBiT monoclonal antibody (1:200) at 4°C overnight, followed by three PBS washes and incubation with Alexa Fluor 488–conjugated goat anti-mouse IgG for 1 h. Images were visualized using a Leica laser scanning confocal microscope.
