# Supplementary material for: Targeted NanoBiT Screening Identifies a Novel Interaction Between SNAPIN and Influenza A Virus M1 Protein
Source: Biology (Basel). 2025 Dec 11;14(12):1770. doi: 10.3390/biology14121770 (PMC12730228; doi:10.3390/biology14121770)
Supplement: Supplementary file 1 [file biology-14-01770-s001.zip › Supplementary Figure S2.pdf]

Supplementary Figure S2

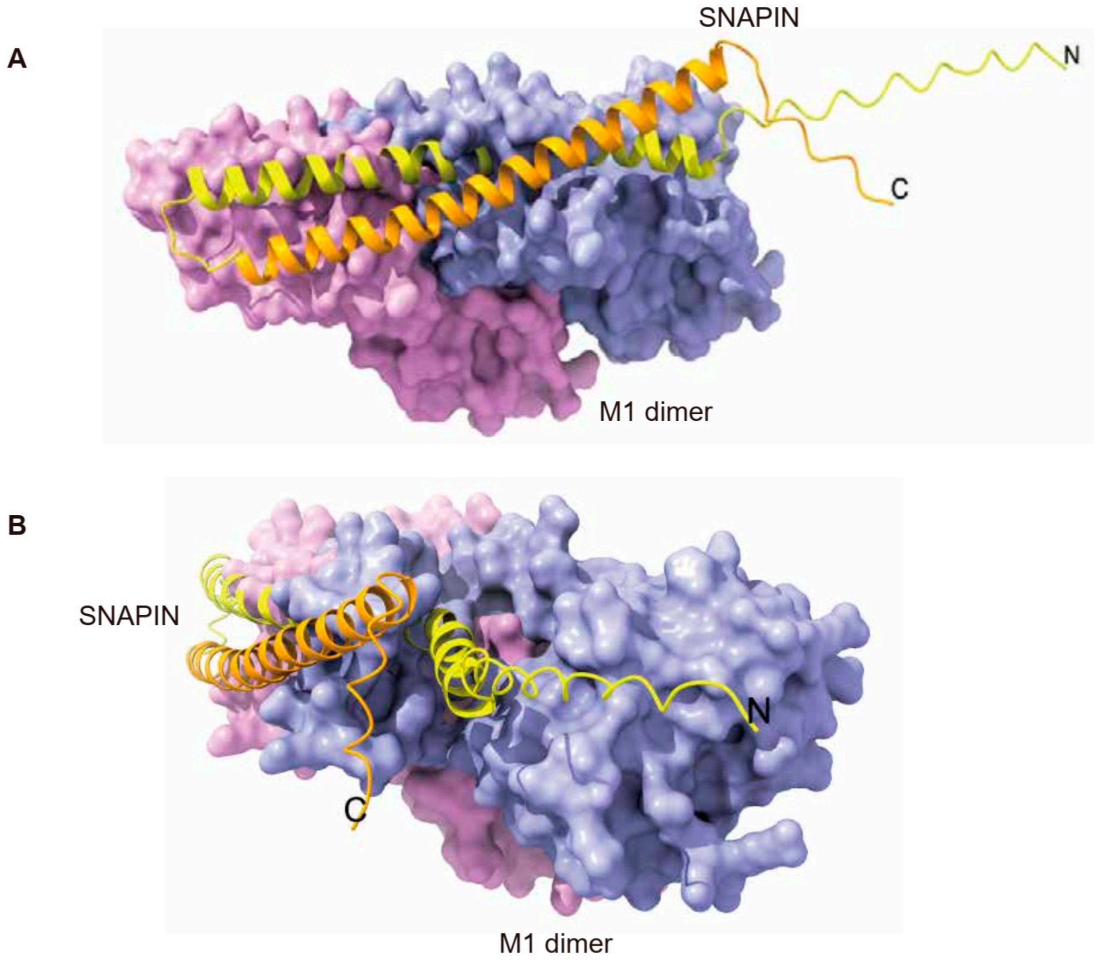

Supplementary Figure S2. Predicted structure of the potential interacting domain between human SNAPIN and IAV M1 protein. Front view (A) and side view (B) of the predicted complex structure.
