# Supplementary material for: Targeted NanoBiT Screening Identifies a Novel Interaction Between SNAPIN and Influenza A Virus M1 Protein
Source: Biology (Basel). 2025 Dec 11;14(12):1770. doi: 10.3390/biology14121770 (PMC12730228; doi:10.3390/biology14121770)
Supplement: Supplementary file 1 [file biology-14-01770-s001.zip › Supplementary Figure S3.pdf]

Supplementary Figure S3

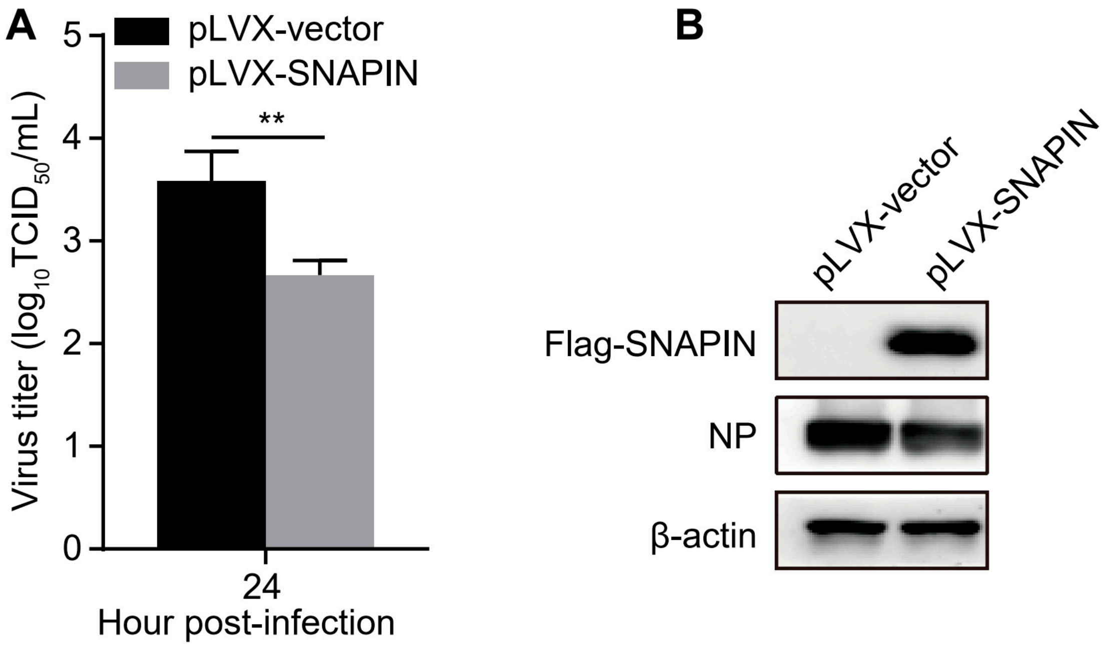

Supplementary Figure S3. SNAPIN suppressed WSN virus replication. A549-Flag-SNAPIN cells infected with WSN virus at an MOI of 1. Virus-containing culture supernatant was collected at 24 hpi and titrated in MDCK cells (A). NP and Flag-SNAPIN proteins were tested by Western blotting (B).
