# Supplementary figures and images for: Targeted NanoBiT Screening Identifies a Novel Interaction Between SNAPIN and Influenza A Virus M1 Protein
Source: Biology (Basel). 2025 Dec 11;14(12):1770. doi: 10.3390/biology14121770 (PMC12730228; doi:10.3390/biology14121770)

Raw data of Figure 2

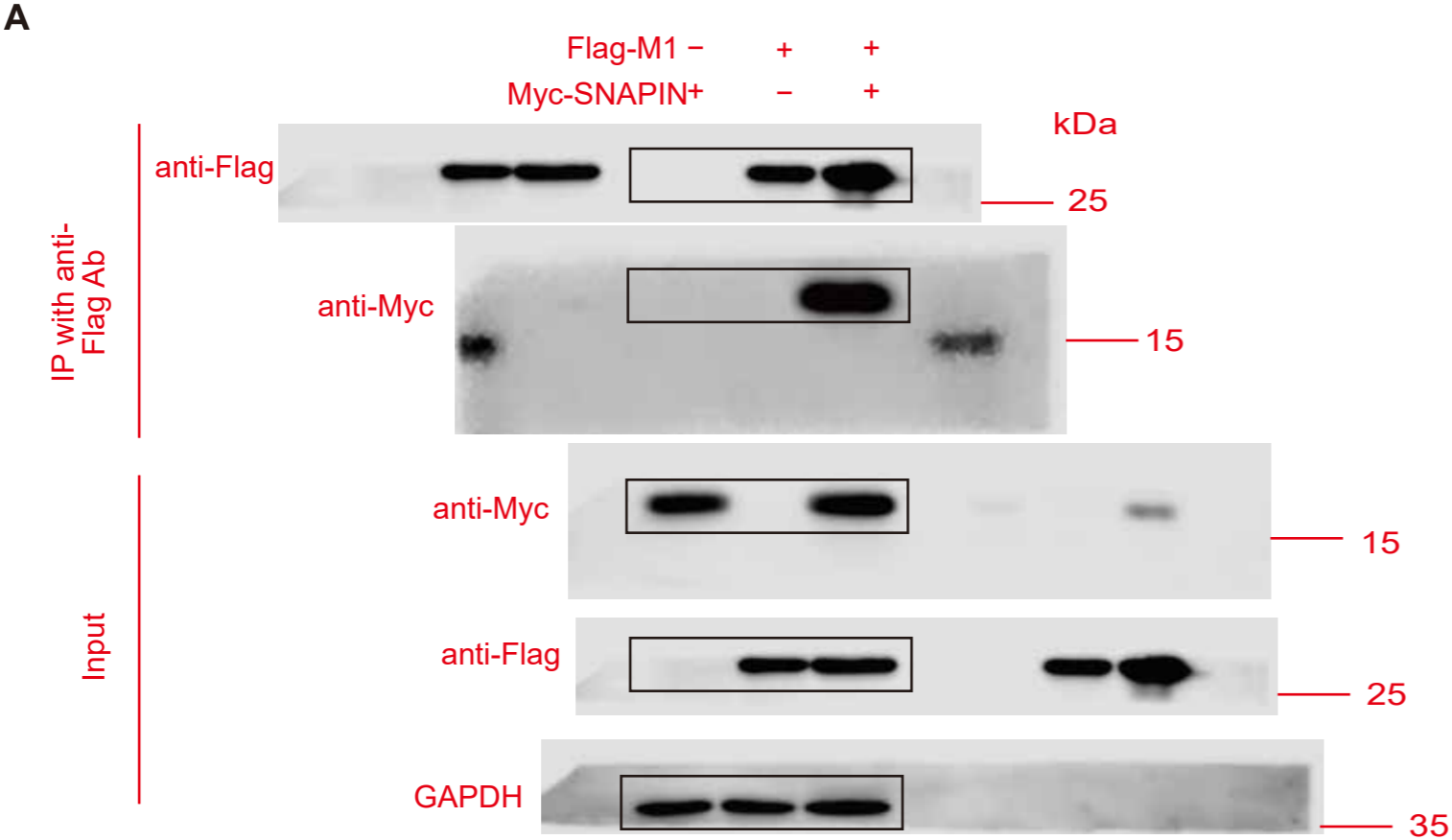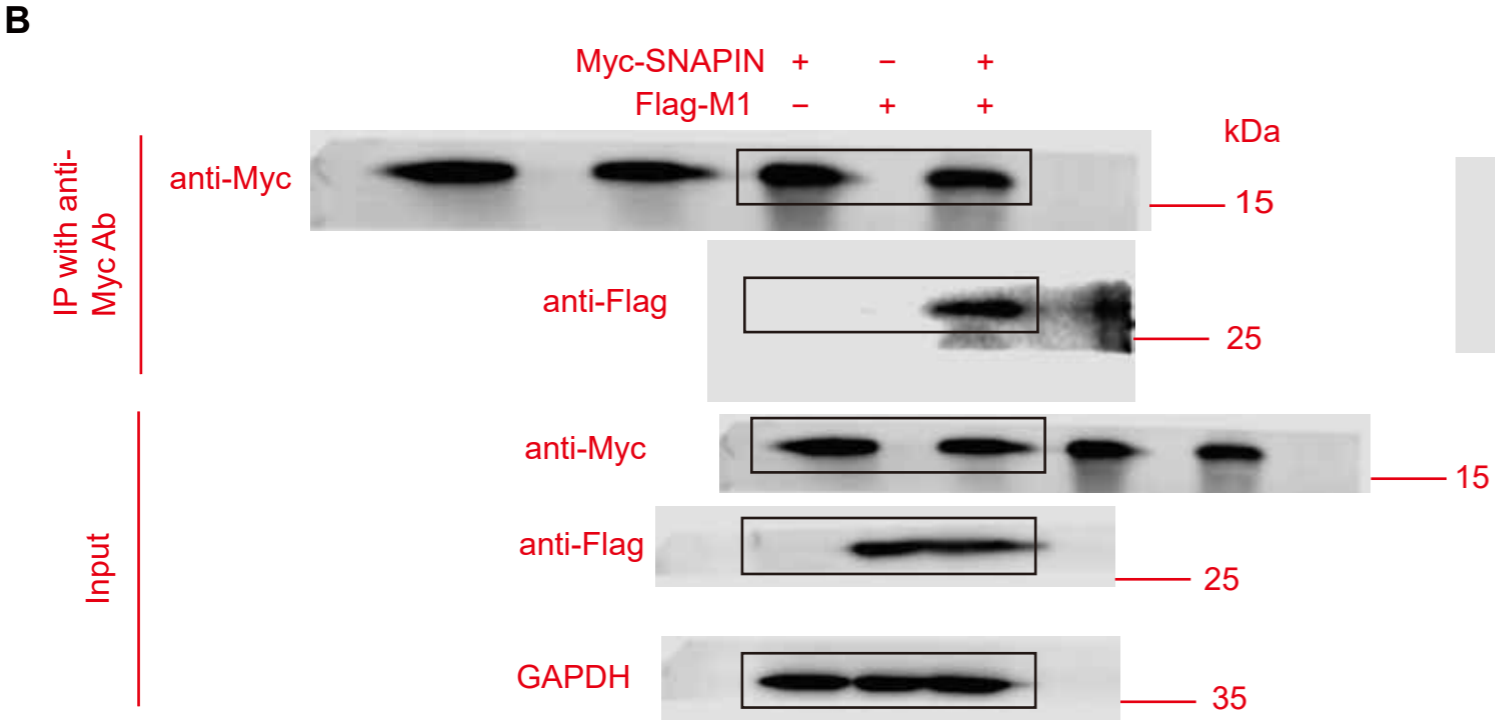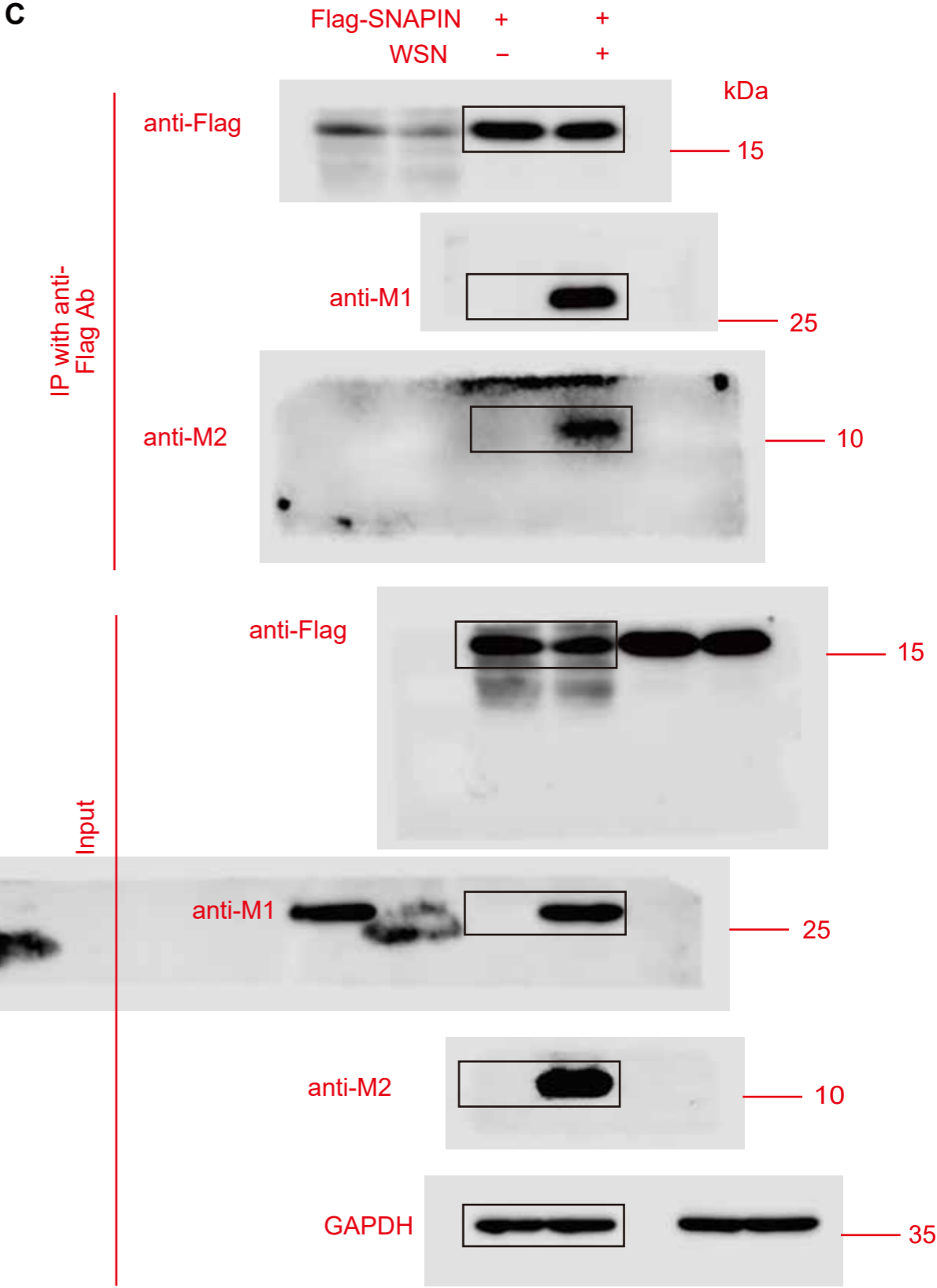

Supplement: Supplementary file 1 [file biology-14-01770-s001.zip › Supplementary Figure S4. Raw data of Figure 2.pdf]

Raw data of Figure 3

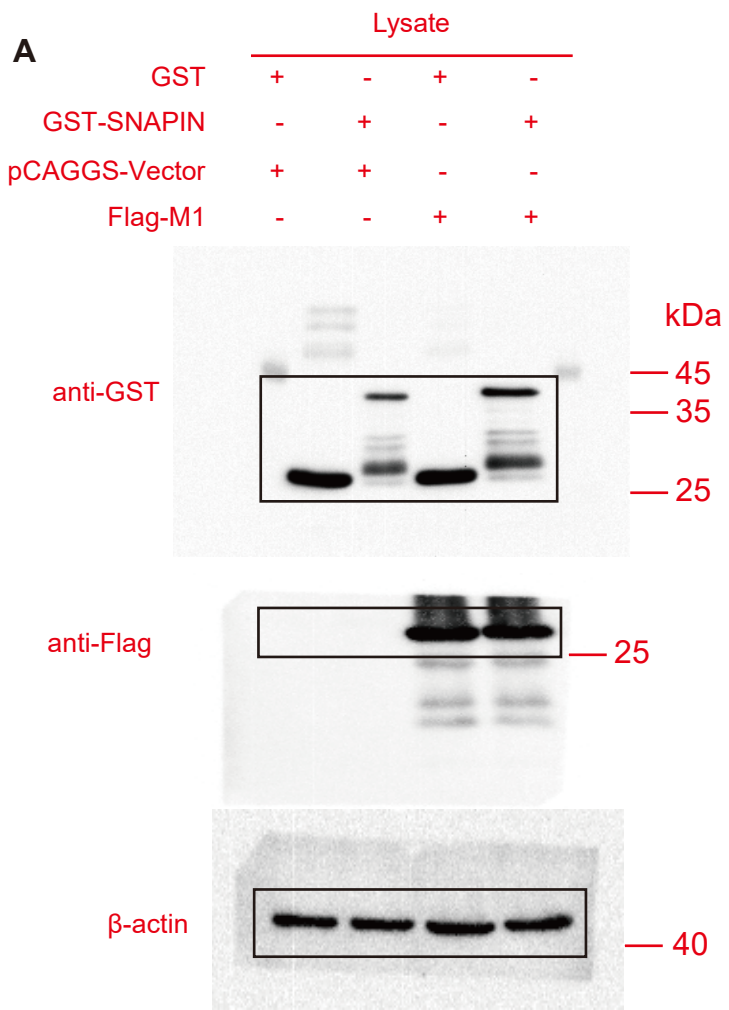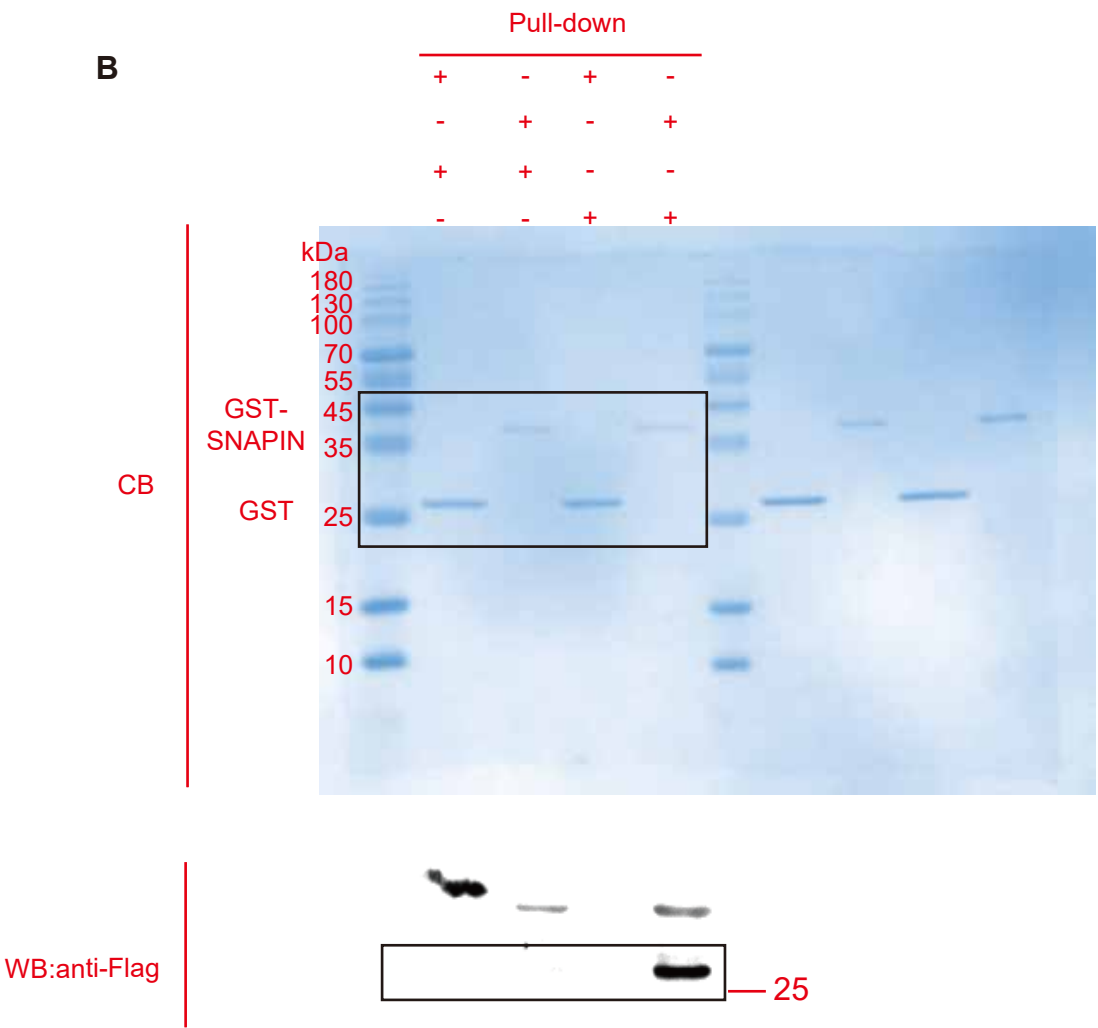

Supplement: Supplementary file 1 [file biology-14-01770-s001.zip › Supplementary Figure S5. Raw data of Figure 3.pdf]
